# Pain patterns and descriptions in patients with radicular pain: Does the pain necessarily follow a specific dermatome?

**DOI:** 10.1186/1746-1340-17-9

**Published:** 2009-09-21

**Authors:** Donald R Murphy, Eric L Hurwitz, Jonathan K Gerrard, Ronald Clary

**Affiliations:** 1 Rhode Island Spine Center, 600 Pawtucket Ave, Pawtucket, RI 02860-6059, USA; 2 Department of Community Health, Alpert Medical School of Brown University, Box G-A, Providence, RI 02912, USA; 3 Department of Research, New York Chiropractic College, 2360 State Route 89, Seneca Falls, New York 13148, USA; 4 Department of Public Health Sciences, John A. Burns School of Medicine, University of Hawaii, Manoa, Hawaii 96822, USA; 5 Aquarius Chiropractic, #210 - 179 Davie Street, Vancouver, V6Z 2Y1, Canada; 6 Private Practice of Chiropractic Medicine, 621 Smith Street, Providence, RI 02908, USA

## Abstract

**Background:**

It is commonly stated that nerve root pain should be expected to follow a specific dermatome and that this information is useful to make the diagnosis of radiculopathy. There is little evidence in the literature that confirms or denies this statement. The purpose of this study is to describe and discuss the diagnostic utility of the distribution of pain in patients with cervical and lumbar radicular pain.

**Methods:**

Pain drawings and descriptions were assessed in consecutive patients diagnosed with cervical or lumbar nerve root pain. These findings were compared with accepted dermatome maps to determine whether they tended to follow along the involved nerve root's dermatome.

**Results:**

Two hundred twenty-six nerve roots in 169 patients were assessed. Overall, pain related to cervical nerve roots was non-dermatomal in over two-thirds (69.7%) of cases. In the lumbar spine, the pain was non-dermatomal in just under two-thirds (64.1%) of cases. The majority of nerve root levels involved non-dermatomal pain patterns except C4 (60.0% dermatomal) and S1 (64.9% dermatomal). The sensitivity (SE) and specificity (SP) for dermatomal pattern of pain are low for all nerve root levels with the exception of the C4 level (Se 0.60, Sp 0.72) and S1 level (Se 0.65, Sp 0.80), although in the case of the C4 level, the number of subjects was small (n = 5).

**Conclusion:**

In most cases nerve root pain should not be expected to follow along a specific dermatome, and a dermatomal distribution of pain is not a useful historical factor in the diagnosis of radicular pain. The possible exception to this is the S1 nerve root, in which the pain does commonly follow the S1 dermatome.

## Background

Radiculopathy in the cervical and lumbar spine is commonly encountered in clinical practice, however, valid population-based estimates are scarce because few non-clinical studies have used valid and reliable diagnostic criteria to detect true nerve root pain [1]. In two studies that used strict criteria, lifetime prevalence of radiculopathy due to a herniated lumbar disk was 4% in females and 5% in males [2,3]. The 2 most common causes of radiculopathy are lateral canal stenosis (LCS) and herniated disk (HD) [4-8]. LCS results from osteophyte formation, or hypertrophied zygapophyseal joints and/or ligamentum flavum. HD results from herniation of nuclear material outside the confines of the annulus fibrosis. In some cases, LCS and HD are present simultaneously. The mechanism of nerve root pain secondary to LCS and chronic HD is believed to be related to vascular congestion and peri- and intraradicular fibrosis [9,10]. In acute HD, it is thought that the pain is primarily chemical in nature [11,12], although pressure can play a contributing role [13].

"Radiculopathy" is not synonymous with "radicular pain" or "nerve root pain". While it is common for patients with radiculopathy to have nerve root pain, the term "radiculopathy" refers to the whole complex of symptoms that can arise from nerve root pathology, including paresthesia, hypoesthesia, anesthesia, motor loss and pain [14]. The terms "radicular pain" and "nerve root pain" specifically apply to a single symptom - pain - that can arise from one of more spinal nerve roots [14].

Accurate diagnosis of patients with spine-related pain is increasingly being recognized as important in helping clinicians make individual treatment decisions. Precise diagnosis can often be elusive, however. Various authors have attempted to investigate improved methods of classifying or diagnosing patients with spine related pain [15-17]. Traditionally, it has been widely held that accurate diagnosis is derived from a combination of history taking, physical examination and special tests. The patient's description of the location and nature of the pain is believed to be an important component of history taking. Pain drawings are often used for this purpose [18,19].

Patients who have spinal pain may also have pain in the upper or lower extremity. This arises from the phenomenon of "referred pain", in which pain is perceived in a wider area that that of the site of origin. This pain can be categorized as nociceptive, neurogenic or psychologic [20]. It is commonly taught in healthcare institutions, and can commonly be found in articles and textbooks, that nerve root pain typically follows along a specific dermatome and that the identification of nerve root pain can be made in part on this basis [21-28]. Typically, statements such as "radiculopathy, or nerve root compression, and therefore pain and neurologic symptoms should follow a dermatomal distribution" [22] and "radicular pain...causes irritation, which cases ectopic nerve impulses perceived as pain in the distribution of the axon" [21] are not accompanied by references to any studies that specifically gather data that allows one to determine whether or not this is a true statement.

On the other hand, other authors have suggested that nerve root pain does not necessarily follow along a specific dermatome [29-31]. These statements are likewise typically made without reference to data. Recently, experimental study has been carried out that investigates the value of dermatome maps. Bove, et al [32] questioned 25 patients with radicular pain in the lower extremity regarding whether the pain was perceived as being on the skin or deep. They assessed this perception both at rest and during the straight leg raise test. In all cases the pain was reported as deep. These authors suggested that this indicates that the diagnostic value of dermatome maps should be questioned. Anderberg, et al [33] assessed 30 patients with cervical radiculopathy and used selected nerve root block to determine the precise level of nerve root pain. They found only a 28% correlation between location of neurologic deficit/dermatomal distribution of the pain and the involved nerve root. Both these studies had small sample sizes, limiting generalizability of the conclusions.

Therefore, the primary research question investigated in this study is, "Does radicular pain in the cervical or lumbar spine tend to follow along a specific dermatome, as displayed in commonly used dermatome maps?" Secondarily, we sought to determine whether scapula area pain is a common complaint in patients with cervical radicular pain. Finally, we sought to determine whether the quality of pain, as described by the patient, is consistent across patients with cervical or lumbar radicular pain and is useful in diagnosis. We set out to investigate these questions by assessing the pain drawings and verbal descriptions of pain location and quality of consecutive patients diagnosed with cervical or lumbar radicular pain. The diagnosis of nerve root pain was made on the basis of reproduction of pain with known reliable and valid nerve root pain provocation procedures (see Methods section for details) and the localization of the nerve root(s) involved was made with imaging and/or electromyography. The descriptions were compared with established dermatome maps to determine whether or not the pain patterns followed along a specific dermatome. In addition, the frequency of the presence of scapula area pain in patients with cervical radicular pain was determined, as well as the relative frequency of various pain descriptors was determined.

## Methods

The methodology was reviewed and approved by the Institutional Review Board at the New York Chiropractic College. The subject population was those patients seen at the Rhode Island Spine Center that fit the criteria for having radicular pain. History and examination was performed by one of two chiropractic physicians, one of whom (DRM) also performed the retrospective chart review of the pain patterns and descriptions (see below) for the purpose of this study. However, all history and examination procedures were performed before the study idea was developed.

Inclusion criteria were:

Age over 18

Ability to communicate well in English

Extremity pain clinically determined by the treating chiropractic physician, using the criteria (see below), to arise from one or more nerve roots.

The criteria for the identification of nerve root pain were:

Disk protrusion, LCS or both clearly demonstrated on appropriate imaging (MRI, CT) or;

EMG documentation of nerve root dysfunction and;

Reliable and valid nerve root provocation tests that exactly reproduce the patient's extremity pain

In addition, neurologic examination included assessment of sensation to pin prick, muscle stretch reflexes ("deep tendon reflexes") and motor strength. This part of the examination does not identify nerve root pain *per se *but can be helpful in localizing the nerve root of involvement [34].

Patients were excluded if their pain was not exactly reproduced by nerve root provocation maneuvers or if their pattern of pain was not clearly drawn or described in the chart.

The examination included (with one exception) pain provocation tests with known reliability and validity for identifying nerve root pain (see Table 1). These tests are designed to stretch the nerve root or increase or decrease pressure on the nerve root. In the case of tests that apply stretch to the nerve root, "structural differentiation" [35,36] maneuvers are used to increase the specificity of the test. A full discussion of the examination for nerve root pain is beyond the scope of this paper and can be found elsewhere [30,35].

**Table 1:** Pain provocations maneuvers used to identify nerve root pain.

**Test**	**Procedure**	**Structural Differentiation**	**Response**
Brachial Plexus Tension Test	The patient lies supine and the scapula is depressed inferiorward. The shoulder is abducted to 90 degrees. The wrist and fingers are extended, the forearm is supinated, the shoulder is externally rotated and the elbow is extended	Elevation of the scapula, ipsilateral lateral flexion of the head, flexion of the wrist and fingers	Reproduction of pain with the procedure, reduction of pain with structural differentiation
Cervical Compression Test (Spurling's test)	The patient is seated. The head is laterally flexed toward the side of symptoms and slightly extended. Downward pressure is applied to the top of the head	None	Reproduction of pain
active cervical rotation	The patient is seated and is asked to rotate the head toward the side of symptoms	None	Reproduction of pain
Cervical Distraction Test	The patient is seated. The head is lifted superiorward to distract the cervical spine	None	Relief of pain
Straight Leg Raise test	The patient is supine. The ankle is dorsiflexed and the leg is raised by flexing the hip while the knee is extended	Plantar flexion of the ankle; Well Leg Raise test	Reproduction of pain with the procedure, reduction of pain with structural differentiation
Femoral Nerve Stretch test	The patient is prone. The knee is flexed while the hip and pelvis remain in the neutral position	Flexion and extension of the head	Reproduction of pain with the procedure, reduction of pain with structural differentiation

In the cervical spine the tests used were the Brachial Plexus Tension Test, Cervical Compression Test (Spurling's test), active cervical rotation and the Cervical Distraction Test. This cluster of tests has been demonstrated to accurately identify nerve root pain in the cervical spine [37]. In the lumbar spine, the Straight Leg Raise test (SLR) and the Femoral Nerve Stretch test (FNST) were used. The SLR has been found to have adequate inter-examiner reliability [38,39] and validity [40], especially when combined with structural differentiation maneuvers such as the Well Leg Raise test and ankle dorsiflexion and plantar flexion [35,36,41]. The FNST has been found to have fair inter-examiner reliability [42], but its validity has not been well studied [40]. It should be noted that these tests were used to determine whether the extremity pain was arising from a neural structure. These tests are not capable of identifying the specific nerve root level that is painful or, in the case of the lower extremity tests, that the neural pain is arising from a nerve root or is arising from a lesion peripheral to the nerve root. For this reason, the nerve root level of involvement was identified with MRI, CT and/or EMG. The findings on these tests were interpreted by independent radiologists in the case of MRI and CT or electromyographer (neurologist or physiatrist) in the case of EMG. With regard to MRI or CT, a nerve root level being involved was identified by the presence in the radiologist's report of disc material, osteophyte, ligamentum flavum material or some combination of these encroaching on the lateral recess or lateral canal.

The files of all included subjects were retrospectively reviewed and the following information obtained:

The patient's description of the pain pattern

The patient's pain drawing

The patient's description of the quality of the pain

The imaging findings

The description and drawing of the pain pattern were reviewed by the lead author (DRM) and a 4^th ^year chiropractic intern (either JKG or RC). They were compared with the dermatome maps of 2 reference sources [43,44]. Although these sources are somewhat dated, they were chosen because there were considered authoritative and because all examiners had familiarity with them from previous use during training and with other research projects. It was decided by the two examiners whether the pain pattern as described followed along a specific dermatome or not. For a pain pattern to be deemed dermatomal, the pain must be contained within the area designated in the reference sources as arising from the nerve root involved. If all or part of the pain pattern fell outside the area designated by both reference sources for the involved nerve root, it was designated non-dermatomal. No distance cut-off was used. In cases in which there was more than one nerve root involved based on imaging and/or EMG, the pain had to be contained within the combined patterns of the involved nerve roots to be designated dermatomal. In cases in which there was disagreement between examiners, discussion was undertaken and an agreement reached.

### Statistics

Patients were stratified by location of pain (cervical vs. lumbar) and nerve roots were stratified by level. For each area and all levels, frequencies and percentages of the pain pattern (dermatomal vs. non-dermatomal) and quality of pain (burning, aching, sharp, other) were computed. Frequencies and percentages of scapular pain were computed for patients with cervical radicular pain. Differences in proportions (across area and by level within area) were tested with Chi-square tests. Data on dermatomal *vs *non-dermatomal pattern, scapular pain *vs *non-scapular pain and pain quality were used to construct 2 × 2 tables. Sensitivities (Ses) and specificities (Sps) of a dermatomal pain pattern and pain qualities (with 95% confidence intervals [CIs]) were also computed for each level; Ses and Sps of scapular pain among patients with cervical radicular pain were computed for each cervical level. Data management and statistical analyses were conducted with Microsoft Excel and SAS (version 9.1, Cary, NC).

## Results

Of the 222 consecutive patients diagnosed with radicular pain who were initially assessed, 53 were excluded. The most common reason for exclusion was absence of imaging or EMG (n = 26). The second most common reason for exclusion was absence of extremity pain (n = 21), followed by insufficient pain description (n = 4) and no distinct lateral canal encroachment on imaging (n = 2). No patients were excluded due to inability of examiners to agree on the designation of dermatomal or non-dermatomal pattern.

Two hundred twenty-six nerve roots (94 cervical, 132 lumbar) in 169 patients (70% female) were finally assessed. The most common levels involved were L5 (n = 49), C6 (n = 40), S1 and C7 (n = 37 each) and L4 (n = 28). More than one level of involvement was demonstrated on imaging in 41 (24%) cases. The results of the assessment of the dermatomal *vs*. non-dermatomal pattern of pain are presented in table 2. Overall, pain related to cervical nerve root pain was non-dermatomal in over two-thirds (69.7%) of cases. In the lumbar spine, the pain was non-dermatomal in just under two-thirds (64.1%) of cases. Regarding specific nerve root levels, the majority of cases involved non-dermatomal pain patterns at all levels except C4 (60.0% dermatomal) and S1 (64.9% dermatomal).

**Table 2:** Comparison of dermatomal *vs*. non-dermatomal patterns of radiculopathy at each level of the cervical and lumbar spine.

	**Dermatomal**		**Non-dermatomal**	
	
**Area/nerve root**	**n**	**Percent**	**n**	**Percent**
Cervical	20	30.3	46	69.7
Lumbar	37	35.9	66	64.1

Chi-square *p *= 0.4510				

Cervical levels				

C4	3	60.0	2	40.0
C5	3	25.0	9	75.0
C6	14	35.0	26	65.0
C7	12	32.4	25	67.6

Chi-square *p *= 0.5731				

Lumbar levels				

L2	2	40.0	3	60.0
L3	4	30.8	9	69.2
L4	8	28.6	20	71.4
L5	8	16.3	41	83.7
S1	24	64.9	13	35.1

Chi-square *p *< 0.0001				

Table 3 presents the data on the presence or absence of scapular pain in patients with cervical radicular pain. In the 64 patients with painful cervical nerve roots, 33 (51.6%) reported pain in the scapula area, and 31 (48.4%) did not. In 2 subjects the presence or absence of scapular pain was not accurately reported. There is empirical though not statistical (p = 0.375) evidence of a trend toward increased likelihood of the presence of scapular pain in lower cervical radiculopathy (40% at C4, 45.5% at C5, 46.2% at C6, and 56% at C7). Of the 33 patients who reported the presence of scapular pain, 26 (78.8%) had HD, with or without LCS, while only 7 (21.2%) of those patients who complained of scapular pain had LCS alone. Of the 31 who reported no scapular pain, 17 (54.8%) had HD with or without LCS and 14 (45.2%) had LCS alone.

**Table 3:** The presence of scapular pain amongst patients with cervical radiculopathy.

	**Scapular Pain Present**		**Scapular Pain Absent**	
	
**Area/nerve root**	**n**	**Percent**	**n**	**Percent**
Cervical	33	51.6	31	48.4
				
Cervical levels				
C4	2	40.0	3	60.0
C5	6	45.5	6	50.0
C6	18	46.2	21	53.8
C7	20	55.6	16	44.4

Chi-square *p *= 0. 8314				

Table 4 presents the data regarding the quality of the pain and nerve root levels. The vast majority of patients (85%) described their pain either as "aching" or "sharp". There was no significant difference between these 2 descriptions for any area of the spine or nerve root level.

**Table 4:** Relationship between quality of pain and nerve root level

**Area/Nerve root**	**Burning**		**Aching**		**Sharp**		**Other/** **Not described**	
	**n**	**percent**	**n**	**percent**	**n**	**percent**	**n**	**percent**

Cervical	3	3.9	40	51.9	25	32.5	9	11.7
Lumbar	10	8.4	56	47.1	45	37.8	8	6.7

Chi-square *p *= 0.3389; *p *= 0.3806 with "Other" excluded								

Cervical Levels								

C4	2	28.6	3	42.9	2	28.6	0	0.0
C5	1	6.7	6	40.0	6	40.0	2	13.3
C6	3	5.9	24	47.1	16	31.4	8	15.7
C7	0	0.0	24	63.2	11	28.9	3	7.9

Chi-square *p *= 0.1531; *p *= 0.1243 with "Other" excluded								

Lumbar levels								

L2	2	33.3	2	33.3	2	33.3	0	0.0
L3	2	14.3	7	50.0	4	28.6	1	7.1
L4	7	20.6	17	50.0	8	23.5	2	5.9
L5	9	15.5	27	46.6	19	32.8	3	5.2
S1	3	7.5	15	37.5	18	45.0	4	10.0

Chi-square *p *= 0.7229; *p *= 0.5031 with "Other" excluded								

Additional file 1 presents the sensitivity (Se) and specificity (Sp) of the presence or absence of a dermatomal pattern of pain and quality of pain by nerve root level as well as the presence of absence of scapular pain. In general, the Se and Sp values for dermatomal pattern of pain are low for all nerve root levels with the exception of the C4 level (Se 0.60, Sp 0.72) and S1 level (Se 0.65, Sp 0.80), although in the case of the C4 level, the number of subjects was small (n = 5). For the S1 level, the positive likelihood ratio was 3.25 and the negative likelihood ratio was 0.44. Likewise, the Se and Sp values for the quality of pain and the presence or absence of scapular pain are low, with the exception of the Sp for the description of "burning" pain (0.86-1.00).

## Discussion

This study failed to find much support for the common notion that extremity pain that arises from radiculopathy typically follows along a specific dermatome. In general, the Se and Sp of this finding were low, suggesting that this factor is not useful in making the diagnosis of radicular pain. The one exception is S1 radicular pain, in which a dermatomal pattern of pain was found in nearly two-thirds of patients and the Se and Sp were high enough (Se 0.65, Sp 0.80) to make this a useful finding in the diagnosis of S1 radiculopathy. In patients with C4 radicular pain, 60.0% had a dermatomal pattern and the Se and Sp were also relatively high (Se 0.60, Sp 0.72), but there were only 5 subjects with radicular pain at this level, so firm conclusions cannot be drawn.

This study does not allow firm conclusions to be drawn about the reason for the absence of a dermatomal pattern of pain in most cases. One of the possibilities for this, however, is that patients with nerve root pain may also have other sources of pain, such as the intervertebral disk, dura mater or other tissues, that are producing a nociceptive, as opposed to neurogenic, pain pattern [20]. Also, as Bove, et al [32] pointed out, it has been demonstrated that spontaneous activity in neurons that innervate muscle or other deep tissues can develop after nerve injury [45] or nerve inflammation [46]. If a portion of the referred limb pain was arising from this spontaneous activity, the pattern of pain would not be expected to follow a specific dermatome. Another possibility is that there can be overlap between dermatomes, with one dermatome encompassing one or two adjacent segments. [47,48] So it may be possible for an individual with nerve root pain to have a dermatomal distribution, but for this distribution to fail to precisely match the pattern depicted in the classic dermatome maps. Finally, it is known that intense and/or persistent nociceptive input can produce an expansion in the size of the receptive fields of those dorsal horn cells that receive and project nociceptive signals from the periphery [49]. As a result, these cells are capable of responding to input from a greater number of incoming afferent fibers, leading to referral of pain that is perceived in a wider area than would occur without this expansion. Nonetheless, none of these factors changes the primary conclusion of this study, i.e., that the dermatome maps commonly used to identify the expected pattern of radicular pain are not useful as a clinical diagnostic tool. Finally, in a patient with conjoined nerve roots, which can be seen on imaging in approximately 4% of individuals [50], the pain may follow the path of both nerve roots, and thus not conform to the dermatome pattern of a single nerve root. None of the patients in this sample had this anomaly, and beside this, multiple nerve root involvement was considered in our analysis.

The findings of this study are consistent with those of other authors. Nitta, et al [51] used selected nerve root block in 71 patients with lumbar radiculopathy and found that nerve root pain at L4 and L5 commonly deviated from the classic dermatomal pattern, but that at S1 typically followed the classic S1 distribution. Bove, et al [32] assessed 25 patients diagnosed with lumbar radiculopathy to determine whether the pain was perceived as "deep" or "on the skin". In all cases the pain was reported to be "deep", both at rest and when evoked by performing a SLR [32]. They concluded that the diagnostic utility of dermatomal maps should be questioned on the basis that in no case was pain described as "on the skin", which would be expected if the pain pattern was dermatomal in nature. Unfortunately, the subjects in the present study were not asked about the superficial *vs*. deep location of their pain, so no confirmation of the finding of Bove, et al [32] could be made. However, it is significant that the conclusions regarding the diagnostic utility of dermatome maps were the same in these two studies. Ljunggren, et al [52] assessed 77 subjects with "lumbago sciatica" secondary to herniated disk and found some similarity in the pain location between patients with L5 those with S1 radiculopathy, but specific dermatomal maps were not used in this comparison. Anderberg, et al [33] found no relationship between the distribution of pain and the level of cervical radicular pain as determined by selective nerve root block.

The dermatome pattern for the S1 nerve root that is most commonly described in the literature involves the posterolateral thigh and leg and the lateral foot. This study found that this pattern of pain was seen in 65% of patients with S1 radicular pain. Thus, a dermatomal pain pattern may be useful diagnostically in patients with S1 nerve root pain. However, it should be noted that no patients who did not have radiculopathy were included in these data. It is known that the lower extremity referred pain pattern of somatic structures innervated by the S1 segment also commonly follows the classic S1 dermatome [53]. In addition, the study did not query subjects as to whether their pain was perceived as deep or superficial. Further work, specifically which assesses how common it is for patients with other pain sources to report pain that follows a similar pattern as that of S1 radiculopathy, is required to clarify this.

For patients with radiculopathy at levels other than S1, the patient's description and drawing of the pain pattern does not appear to be a useful piece of diagnostic information. Clinicians should not expect the pain from radiculopathy at levels other than S1 to follow along a specific dermatome.

Scapular pain was present in approximately half the patients with cervical radicular pain. There was a trend toward increased likelihood of the presence of scapular pain relative to nerve root level, suggesting that the lower the cervical nerve root of involvement, the greater the likelihood of the presence of scapular pain. However, the small sample size does not allow definitive conclusions to be drawn about this. It is not clear whether the scapular pain arises from the nerve root itself or from other sources of pain in these patients. However, it is interesting that a strong majority (78.8%) of those patients who reported scapular pain had HD, with or without LCS. The commonness of scapular pain in patients with HD may suggest that the scapular pain may arise from referred pain from the disk itself, rather than arising from the nerve root. Slipman, et al [54] assessed the referred pain patterns of 41 patients undergoing provocative discography in the cervical spine. They found that the scapula area was one of the most common areas of referred pain in these patients, and was reported most commonly by patients with concordant pain provoked by injection of the C4-5 through C6-7 levels. This is consistent with the findings presented here that scapular pain was most common in patients with nerve root pain from C5, C6 and C7 which, in those cases in which HD was present, would involve the C4-5 through C6-7 levels. However, additional work in the area of sources of referred scapular pain is required before firm conclusions can be drawn. In addition, because of the low Se and SP, the presence of scapular pain is not useful for the purpose of diagnosing nerve root pain *per se*. Further work is needed to determine the diagnostic utility of the presence of scapular in diagnosing disk pain.

The majority of patients described the quality of their pain as either "aching" or "sharp". Far fewer described the pain as "burning". There were no significant differences between nerve root levels with regard to pain description. The Se and Sp for "aching" and "sharp" pain descriptions were low, suggesting that these descriptions are of little diagnostic value in identifying nerve root pain. It appears from the data presented here that the description of "burning" pain is highly specific (Sp 0.86-1.00) for the presence of radicular pain, however, given the low number of positive responses to this description, these high estimates of specificity are likely an artifact of the study population and require confirmation in other clinical populations.

One potential weakness of this study is its retrospective nature. However, this may also be seen as a strength in that the description of each patient's pain pattern was recorded by the examining clinician in the manner that is normally carried out in clinical practice, rather than as part of a research project on the dermatomal or non-dermatomal nature of nerve root pain. Thus, clinician bias regarding the expected pain pattern was not a factor in this recording.

## Conclusion

It is concluded from the data presented here that in most cases nerve root pain should not be expected to follow along a specific dermatome, at least as described by commonly used dermatomal maps, and a dermatomal distribution of pain is not a useful historical factor in the diagnosis of radiculopathy. The exception to this is S1 radicular pain, in which the pain does commonly follow the S1 dermatome. Scapular pain is common in patients with cervical radicular pain, particularly those whose nerve root pain is related to HD, and may represent referred pain from the disk itself. The quality of pain is generally an insensitive and non-specific finding in patients with nerve root pain.

## Competing interests

The authors declare that they have no competing interests.

## Authors' contributions

DRM originally conceived of the study served as an examiner. He was also the main writer of the manuscript. ELH was responsible for statistical analysis and writing and editing the manuscript. JKG and RC served as examiners, assisted with literature review, and took part in writing the manuscript. All authors read and approved the final manuscript.

## Supplementary Material

Additional file 1 **Sensitivity and specificity data**. Sensitivity and specificity of the presence or absence of a dermatomal pattern of pain and quality of pain by nerve root level. Click here for file
