# Supplementary material for: Pain patterns and descriptions in patients with radicular pain: Does the pain necessarily follow a specific dermatome?
Source: Chiropr Osteopat. 2009 Sep 21;17:9. doi: 10.1186/1746-1340-17-9 (PMC2753622; doi:10.1186/1746-1340-17-9)
Supplement: Additional file 1 — Sensitivity and specificity data. Sensitivity and specificity of the presence or absence of a dermatomal pattern of pain and quality of pain by nerve root level. [file 1746-1340-17-9-S1.DOC]

C4 C5 C6 C7 L2 L3 L4 L5 S1

________________________________________________________________________________________________________________________________

Dermatome

Se 0.60 0.25 0.35 0.32 0.40 0.31 0.29 0.16 0.65

(95% CI) (0.15, 0.95) (0.055, 0.57) (0.21, 0.52) (0.18, 0.50) (0.053, 0.85) (0.091, 0.61) (0.13, 0.49) (0.073, 0.30) (0.48, 0.80)

Sp 0.72 0.69 0.77 0.72 0.64 0.63 0.61 0.46 0.80

(95% CI) (0.59, 0.83) (0.55, 0.81) (0.56, 0.91) (0.53, 0.87) (0.54, 0.74) (0.53, 0.73) (0.49, 0.72) (0.33, 0.60) (0.69, 0.89)

Burning pain

Se 0.40 0.083 0.079 0.00 0.40 0.15 0.25 0.18 0.08

(95% CI) (0.053, 0.85) (0.002, 0.38) (0.016, 0.21) (0.00, 0.097) (0.053, 0.85) (0.019, 0.46) (0.11, 0.45) (0.088, 0.32) (0.017, 0.22)

Sp 0.98 0.96 1.0 0.89 0.90 0.90 0.93 0.94 0.86

(95% CI) (0.91, 0.99) (0.87, 0.99) (0.86, 1.0) (0.71, 0.98) (0.82, 0.95) (0.81, 0.95) (0.85, 0.98) (0.85, 0.99) (0.76, 0.94)

Ache

Se 0.60 0.50 0.63 0.69 0.40 0.54 0.61 0.55 0.41

(95% CI) (0.15, 0.95) (0.21, 0.79) (0.46, 0.78) (0.52, 0.84) (0.053, 0.85) (0.25, 0.81) (0.41, 0.79) (0.40, 0.69) (0.25, 0.58)

Sp 0.36 0.33 0.36 0.44 0.47 0.48 0.51 0.50 0.41

(95% CI) (0.24, 0.50) (0.21, 0.48) (0.18, 0.58) (0.26, 0.64) (0.37, 0.57) (0.37, 0.59) (0.39, 0.62) (0.36, 0.64) (0.29, 0.54)

Sharp pain

Se 0.40 0.50 0.42 0.33 0.40 0.31 0.29 0.39 0.49

(95% CI) (0.053, 0.85) (0.21, 0.79) (0.26, 0.59) (0.19, 0.51) (0.053, 0.85) (0.091, 0.61) (0.13, 0.49) (0.25, 0.54) (0.32, 0.66)

Sp 0.59 0.61 0.60 0.48 0.56 0.54 0.51 0.52 0.59

(95% CI) (0.45, 0.71) (0.46, 0.74) (0.39, 0.79) (0.29, 0.68) (0.46, 0.66) (0.44, 0.65) (0.39, 0.62) (0.38, 0.66) (0.46, 0.71)

Scapular pain

Se 0.40 0.50 0.46 0.56

(95% CI) (0.053, 0.85) (0.21, 0.79) (0.30, 0.63) (0.38, 0.72)

Sp 0.48 0.48 0.40 0.54

(95% CI) (0.34, 0.61) (0.34, 0.62) (0.21, 0.61) (0.34, 0.73)
